# A combination of joint linkage and genome-wide association study reveals putative candidate genes associated with resistance to northern corn leaf blight in tropical maize

**DOI:** 10.3389/fpls.2024.1448961

**Published:** 2024-10-03

**Authors:** Noel Ndlovu, Manje Gowda, Yoseph Beyene, Biswanath Das, Suresh L. Mahabaleswara, Dan Makumbi, Veronica Ogugo, Juan Burgueno, Jose Crossa, Charles Spillane, Peter C. McKeown, Galina Brychkova, Boddupalli M. Prasanna

**Affiliations:** ^1^ Global Maize Program, International Maize and Wheat Improvement Center (CIMMYT), Nairobi, Kenya; ^2^ Agriculture & Bioeconomy Research Centre, Ryan Institute, University of Galway, Galway, Ireland; ^3^ Biometrics and Statistics Unit, International Maize and Wheat Improvement Center (CIMMYT), Texcoco, Estado. de México, Mexico

**Keywords:** northern corn leaf blight (NCLB), genome-wide association study (GWAS), quantitative trait locus (QTL) mapping, genomic selection (GS), tropical maize, sub-Saharan Africa (SSA)

## Abstract

Northern corn leaf blight (NCLB), caused by *Setosphaeria turcica*, is a major fungal disease affecting maize production in sub-Saharan Africa. Utilizing host plant resistance to mitigate yield losses associated with NCLB can serve as a cost-effective strategy. In this study, we conducted a high-resolution genome-wide association study (GWAS) in an association mapping panel and linkage mapping with three doubled haploid (DH) and three F_3_ populations of tropical maize. These populations were phenotyped for NCLB resistance across six hotspot environments in Kenya. Across environments and genotypes, NCLB scores ranged from 2.12 to 5.17 (on a scale of 1–9). NCLB disease severity scores exhibited significant genotypic variance and moderate-to-high heritability. From the six biparental populations, 23 quantitative trait loci (QTLs) were identified, each explaining between 2.7% and 15.8% of the observed phenotypic variance. Collectively, the detected QTLs explained 34.28%, 51.37%, 41.12%, 12.46%, 12.11%, and 14.66% of the total phenotypic variance in DH populations 1, 2, and 3 and F_3_ populations 4, 5, and 6, respectively. GWAS, using 337,110 high-quality single nucleotide polymorphisms (SNPs), identified 15 marker–trait associations and several putative candidate genes linked to NCLB resistance in maize. Joint linkage association mapping (JLAM) identified 37 QTLs for NCLB resistance. Using linkage mapping, JLAM, and GWAS, several QTLs were identified within the genomic region spanning 4 to 15 Mbp on chromosome 2. This genomic region represents a promising target for enhancing NCLB resistance via marker-assisted breeding. Genome-wide predictions revealed moderate correlations with mean values of 0.45, 0.44, 0.55, and 0.42 for within GWAS panel, DH pop1, DH pop2, and DH pop3, respectively. Prediction by incorporating marker-by-environment interactions did not show much improvement. Overall, our findings indicate that NCLB resistance is quantitative in nature and is controlled by few major-effect and many minor-effect QTLs. We conclude that genomic regions consistently detected across mapping approaches and populations should be prioritized for improving NCLB resistance, while genome-wide prediction results can help incorporate both major- and minor-effect genes. This study contributes to a deeper understanding of the genetic and molecular mechanisms driving maize resistance to NCLB.

## Introduction

As a staple crop in sub-Saharan Africa (SSA), maize underpins food security and livestock production while also providing a vital source of income for smallholder farmers. This crop contributes no less than 30% of the aggregate caloric intake for people in this region (Nuss and Tanumihardjo, 2010). In this context, initiatives focusing on enhancing productivity and sustainability hold the potential to empower maize-dependent smallholder farming communities. Although advances in agricultural research and technology have resulted in a gradual increase in maize production, average maize field productivity in SSA (∼1.7 tons/ha) is far below the global average (∼5 tons/ha) (Prasanna et al., 2020). This can be attributed to various abiotic and biotic stressors, including low soil nitrogen (Das et al., 2019; Ndlovu et al., 2022; Kimutai et al., 2023), inadequate availability and use of farm inputs (e.g., improved seed and fertilizer), insect pest infestations (Kansiime et al., 2023), and disease incidences. Some of these stresses have a negative effect on grain quality or nutrient composition (Sharma and Carena, 2016; Ndlovu et al., 2024a), with far-reaching implications for household nutrition in smallholder communities. Most importantly, diseases such as gray leaf spot (GLS) (Lehmensiek et al., 2001; Benson et al., 2015; Kibe et al., 2020a), maize lethal necrosis (MLN) (De Groote et al., 2016; Beyene et al., 2017; Boddupalli et al., 2020), and northern corn leaf blight (NCLB) (Ding et al., 2015; Chen et al., 2016; Galiano-Carneiro et al., 2021) have been reported to cause considerable losses in maize production systems. The latter has been widely observed to significantly reduce maize grain yield on a global scale, as reported in earlier studies (De Rossi et al., 2022; Raymundo and Hooker, 1981).

NCLB (also known as *Turcicum* leaf blight) is a foliar disease of maize caused by the ascomycete fungus *Setosphaeria turcica* (anamorph *Exserohilum turcicum*) (Li et al., 2018; Jindal et al., 2019; Cao et al., 2020; Ahangar et al., 2022). *Setosphaeria turcica* is categorized into distinct races distinguished by their virulence levels against *Ht* (*Helminthosporium turcicum*) genes in maize (Galiano-Carneiro and Miedaner, 2017). *Ht* genes are recognized for providing race-specific qualitative resistance that is inherited through single genes (Bentolila et al., 1991; Zaitlin et al., 1993; Yin et al., 2003; Xiao et al., 2007; Chung et al., 2010). However, environmental factors may influence the expression of the *Ht* genes within maize or the avirulence genes of *Setosphaeria turcica*, leading to resistance that is unstable and/or less durable (Rashid et al., 2020). NCLB is commonly widespread in mid-altitude tropical and sub-tropical regions with moderate temperatures (17°C–28°C) and cloudy weather coupled with high rainfall and high humidity (Hooda et al., 2017; Bankole et al., 2023). Tropical regions show high pathogen abundance and genetic diversity, resulting in inflated disease severity and a high risk of resistance breakdown (Rashid et al., 2020). These favorable conditions allow the fungus to spread biotrophically during the initial infection process and later switch to a necrotrophic lifestyle. Infections manifest as lesions on leaves and necrosis, leading to a drastic reduction in the photosynthetic potential of the host plant, leading to huge grain yield losses (Ramathani et al., 2011; Ding et al., 2015). The disease affects maize at all growth stages, and infection at an early stage leads to premature death and reduced vigor (Welz and Geiger, 2000; Muiru et al., 2007).

The key to tackle NCLB in tropical maize lies in uncovering the pathogen’s mechanism of infection and the factors that govern resistance. Wisser et al. (2006) alluded that understanding the genetic architecture of host resistance is the foundation of disease management. This is also highly relevant for resistance breeding programs (Yang et al., 2017). However, despite the modern tools and advancements in this field, the genetic basis of resistance to several foliar diseases (like NCLB) in maize is still not well understood. Several studies focusing on the genetics of NCLB resistance documented a combination of both qualitative and quantitative types of inheritance (Welz and Geiger, 2000; Poland et al., 2011; Li et al., 2018). Qualitative resistance stems from allelic variations at only one or two resistance genes, with allele effects large enough to reliably deduce a plant’s resistance genotype from its phenotype (St. Clair, 2010), irrespective of environmental fluctuations (Yang et al., 2017). Quantitative resistance, which is widely used by maize breeders (Yang et al., 2017), refers to the contribution of several genes with small additive effects for the expressed resistance. Resistance to NCLB has been reported to be governed by genes with additive effects coming from a few major genes (such as *Ht1*, *Ht2*, *Ht3*, *ht4*, *Htnl*, *Html*, and *NN*) (Welz and Geiger, 2000; Kaefer et al., 2017; Li et al., 2018). Earlier breeding schemes have predominantly relied on qualitative resistance governed mainly by major gene effects, conferred by *Ht genes*, which are vulnerable to break down of resistance and are impeded by the emergence of new races of the pathogen through recombination events or mutation (Wende et al., 2018). In tropical settings, a broad-based quantitative resistance to NCLB is preferred. This can be achieved either through quantitative disease resistance loci (dQTLs) independently or in conjunction with potent *Ht* genes (Rashid et al., 2020). Carson and Dyke (1994) alluded that quantitative resistance is more durable because it includes both major and minor effect quantitative trait loci (QTL). In this respect, for the development of comprehensive resistance to NCLB in maize, a combination of both additive and non-additive effects is required (Ogliari et al., 2007; Vieira et al., 2009).

Several mapping studies have been conducted to identify QTLs associated with NCLB resistance across diverse maize germplasm and environments. Freymark et al. (1993) and Dingerdissen et al. (1996) identified five QTLs, which together explained 48% of total phenotypic variance (PVE) for NCLB resistance in F_2:3_ populations. With a high-density genetic map, Chen et al. (2016) identified a major QTL *qNCLB5.04* (for NCLB disease severity and lesion size), which explained 20% of the observed phenotypic variation. Another study by Omondi et al. (2023) identified 18 QTLs, which together explained 64.9% of the total PVE in NCLB disease severity. Poland et al. (2011) and Li et al. (2018) used populations derived from multiple parents in nested association mapping (NAM) design and identified 29 and 48 NCLB-associated QTLs, respectively. Depending on the type of population used for QTL mapping, researchers reported the presence of both additive and non-additive effects for NCLB resistance in these studies.

Genome-wide association study (GWAS) is another robust and cost-effective method for dissecting polygenic traits, enhancing mapping resolution, and complementing linkage mapping techniques (Gowda et al., 2021). GWAS has been employed to detect allelic variations for MLN (Gowda et al., 2015), GLS (Kibe et al., 2020a), tar spot complex (Cao et al., 2017), corn rust (Wang et al., 2023), sorghum downy mildew (Rashid et al., 2018), and NCLB (Galiano-Carneiro et al., 2021). Several research studies have documented GWAS results on NCLB resistance in maize, predominantly concentrating on temperate germplasm and environments (Rashid et al., 2020). Using a large association mapping panel with 999 inbred lines, Ding et al. (2015) revealed 81 significantly associated SNPs for NCLB resistance. Another study by Van Inghelandt et al. (2012) identified significant SNPs on chromosomes 2, 5, 6, 7, and 9. Although numerous QTLs that control resistance to NCLB have been identified in maize, the current understanding of the genetic architecture particularly in the germplasm adapted to SSA is low. Indeed, GWAS and linkage analyses have their own merits and demerits when used independently. For example, because of population structure, GWAS generally shows higher false positive rates compared to linkage analysis (Yu et al., 2006; Zhang et al., 2018). In this respect, methods that incorporate linkage mapping and GWAS can bring together the merits of both approaches. Such combined approaches have been successfully applied to reveal the genetic basis of complex quantitative traits like MLN (Sitonik et al., 2019) and tar spot complex (Cao et al., 2017).

Genomic prediction (GP), previously developed for dairy breeding, has also been used in plant breeding for disease resistance. GP utilizes all available phenotyped and genotyped marker data of a training set to build a prediction model while bypassing the need for QTL detection (Meuwissen et al., 2001). GP has successfully been applied in the prediction of resistance to diseases such as *Gibberella* ear rot (Han et al., 2016), MLN (Gowda et al., 2015, 2018), and Goss’s wilt (Cooper et al., 2019) in maize. However, only a few studies have reported GP in NCLB resistance in maize (Technow et al., 2013; Omondi et al., 2023). GP studies have included genotype-by-environment interactions by performing overall predictions across environments (Heffner et al., 2011), within environments (Burgueño et al., 2012; Heslot et al., 2014), or using marker-by-environment interactions (Jarquín et al., 2014; Lopez-Cruz et al., 2015). In this respect, the objectives of the study were (1) to investigate the phenotypic variation for NCLB resistance and its correlation with other agronomic traits in tropical and sub-tropical maize lines; (2) to use linkage mapping, JLAM, and GWAS to identify genomic regions associated with NCLB resistance; and (3) to assess the potential of utilizing GP in breeding to improve NCLB resistance.

## Materials and methods

### Plant materials and field trials

Here, we evaluated one association mapping (IMAS) panel, three biparental doubled haploid (DH) populations, and three biparental F_3_ populations across diverse environments in Kenya (**Table 1**). The parental lines used to form DH populations (CML494, CML504, CML511, and CML550) and F_3_ populations (CML505, CZL074, CZL009, CZL0723, CZL0719, and LapostaSeqC7-F103-1-2-1-1) showed a wide variation for foliar diseases including NCLB, corn rust, and GLS and known donors for low soil nitrogen stress and drought tolerance. More information on these populations has been reported in earlier studies (Ertiro et al., 2020b; Sitonik et al., 2019). The IMAS panel (composed of 390 inbred lines) was evaluated in replicated trials established across six Kenyan locations (i.e., Alupe 2012, Alupe 2013, Alupe 2014, Kibos 2013, Kibos 2014, and Embu 2013). The field trials were established in disease hotspot locations under natural infestation during the long rainy seasons (March to August). All three locations—Kibos [−0.03861°S, 34.81596°E; 1,193 m above mean sea level (masl); 865-mm mean annual rainfall), Alupe (0.503725°N, 34.12148°E; 1,153 masl; 1,400-mm mean annual rainfall), and Embu (0°31′52″ S 37°27′02″ E, 1,406 masl; 1,206-mm mean annual rainfall)—have a bimodal rainfall distribution. All three DH populations [CML494×CML550 (DH pop 1), CML504 × CML550 (DH pop 2), and CML511 × CML550 (DH pop 3)] were evaluated in Kakamega (0°17′3.19″ N 34°45′8.24″ E, 1,535 masl) and Kitale (1.0191°N 35.0023°E, 1900 masl) for 2 years in 2014 and 2015. Three F_3_ populations (F_3_ pop 4, CZL074 × LaPostaSeqC7-F103-1-2-1-1; F_3_ pop 5, CZL009 × CML505; and F_3_ pop 6, CZL0723 × CZL0719) were evaluated in two locations in Kakamega and Embu in 2013 during long rainy season (**Table 1**; **Supplementary Table S1**). All populations were evaluated in one row of 4-m plots, with two replications. For all these trials, two seeds were planted per hill and thinned to a single plant per hill 3 weeks after emergence. This was done to ensure a uniform plant density per entry. Alpha (α)–lattice experimental design was used, and all standard agronomic practices were applied.

**Table 1:** Trait means, estimates of heritability, and variance components for NCLB disease severity in the IMAS panel, DH, and F_3_ populations evaluated in multiple environments.

Population	# Loc	Pop size	Mean	σ^2^G	σ^2^GE	σ^2^e	*h^2^*
IMAS panel	6	390	4.16	0.19**	0.09**	0.18	0.86
CML494 × CML550 (DH pop 1)	4	110	5.17	0.02*	0.01*	0.15	0.52
CML504 × CML550 (DH pop 2)	4	210	3.64	0.06**	0.02*	0.18	0.68
CML511 × CML550 (DH pop 3)	3	107	4.69	0.17**	0.06**	0.15	0.79
Across three DH pops	4	421	5.24	0.33**	0.03**	0.29	0.88
CZL074 × LaPostaSeqC7-F103-1-2-1-1 (F_3_ pop 4)	2	172	3.93	0.01*	0.01*	0.13	0.25
CZL0009 × CML505 (F_3_ pop 5)	3	195	2.21	0.01*	0.02*	0.07	0.30
CZL0723 × CZL0719 (F_3_ pop 6)	2	195	3.06	0.05*	0.00	0.14	0.59

* and ** indicate significance at *P* < 0.05 and *P* < 0.01, respectively. *σ^2^G*, *σ^2^GxE*, *σ^2^e*, and *h^2^* refer to genotypic variance, genotype × environment interaction variance, error variance, and broad sense heritability, respectively.

### Phenotypic evaluation

The locations used in this study are all hotspots for NCLB. Disease severity data were visually rated based on an ordinal scale of 1 (highly resistant, without disease symptoms) to 9 (highly susceptible, leading to necrosis) (Ding et al., 2015). For each population, each location-year combinations were treated as an independent environment, which resulted in six environments for the IMAS panel, four environments for DH populations, and two environments for each F_3_ population. In addition to the NCLB disease severity score, data were collected for other agronomic traits, including days to anthesis (AD; days from planting to 50% pollen shed), days to silking (SD; days from planting to 50% silking), and anthesis to silking interval (ASI; calculated as the difference between SD and AD). Plant height (PH; measured as the length in centimeters from the base of a plant to the insertion of the first tassel branch of the same plant for 10 representative plants per plot) and ear height (EH; measured as the length in centimeters from the base of a plant to the internode of the top ear of the same plant for 10 representative plants per plot). Ear position (EPO) was calculated as the ratio between PH and EH. GLS disease severity data were recorded at the mid-silking stage and scored plot-wise on an ordinal scale of 1 (highly resistant, without disease symptoms) to 9 (highly susceptible, leading to necrosis). Before harvesting the crop, the number of ears in each plot with portion of the ear exposed was recoded and expressed as a percentage of poor husk cover (HC) relative to the total number of ears harvested. Grain yield (GY) was calculated using the field weight of ears per plot, assuming an 80% shelling percentage and adjusting for a moisture content of 12.5%. Grain texture (TEX) was measured on a 1-to-5 scale (where 1 = flint, 2 = semi-flint, 3 = intermediate, 4 = semi-dent, and 5 = dent). NCLB disease severity data were recorded on an ordinal scale of 1 to 9, and the data met all the assumptions of the applied statistical model (i.e., normally distributed, constant variance, and independent) (Rawlings et al., 1998).

Analysis of variance for individuals and across environments was carried out using the ASREML-R (Gilmour et al., 2015; Kibe et al., 2020a, b) for the IMAS panel and DH and F_3_ populations. The linear mixed model with the restricted maximum likelihood (REML) was used to calculate all variance components. The study treated replication as a fixed effect and all other treatment effects as random. As defined by Knapp et al. (1985), the components of variance were estimated using the complete random effects model, whereas broad-sense heritability was calculated as the ratio of genotypic to PVE. META-R software (Alvarado et al., 2015) was used to generate best linear unbiased predictions (BLUPs) and best linear unbiased estimators (BLUEs), which were used in downstream processes.

### Genotyping-by-sequencing

The IMAS panel and DH populations used in this study have also been used in earlier studies for different traits (Sitonik et al., 2019; Ertiro et al., 2020a; Kibe et al., 2020a). The populations have been routinely used because the lines are representative of tropical and sub-tropical regions, developed over time. For all the inbred lines from the IMAS panel and biparental populations, DNA was extracted, purified, and genotyped with high-density makers using genotyping-by-sequencing (GBS) at the Institute of Genomic Diversity, Cornell University, USA, as described in earlier studies (Elshire et al., 2011; Gowda et al., 2015, 2021; Ndlovu et al., 2022). GBS data for all biparental populations were filtered by using TASSEL version 5.2 (Bradbury et al., 2007) with a criterion of excluding markers with heterozygosity of >5%, minor allele frequency (MAF) of < 0.05, and a minimum count of 90%. Only polymorphic SNPs between the parents and marker loci homozygous for both parents were retained in each biparental population. Finally, SNPs were further filtered with the criteria of minimum distance between adjacent SNPs as ≥200 Kilo base pairs to ensure uniform distribution of markers throughout the genome.

### Linkage mapping

The linkage map for each biparental population was constructed by using QTL IciMapping version 4.1 (Meng et al., 2015). Finally, we used 2,105, 2,699 1,962, 1,160, 1139, and 1,160 high-quality SNPs in DH pop 1, DH pop 2, DH pop 3, F_3_ pop 4, F_3_ pop 5, and F_3_ pop 6, respectively. The linkage map was constructed by using these SNPs and by selecting the most significant markers using stepwise regression. A likelihood ratio test was used to calculate the logarithm of odds (LOD) for each marker at a score of >3 with a 30-cM maximum distance between two loci. The Kosambi mapping function (Kosambi, 1944) was used to transform the recombination frequencies between two linked loci. BLUPs across environments were used to detect QTLs based on inclusive interval mapping for each population. Phenotypic variation explained by individual QTLs and total variation explained by all QTLs together was estimated. QTL naming was done with the letter “q” indicating QTL, followed by an abbreviation of the trait name, the chromosome, and the marker position, respectively. For comparisons of QTL positions detected in different biparental populations, QTL sharing the same flanking markers and within the same chromosome interval were defined as the same QTL.

### Joint linkage association mapping

All three DH populations were genotyped with GBS markers and then combined for filtering. As a first step in quality control, we excluded markers with a heterozygosity of >5%, MAF of < 0.05, and a minimum count of 90%. For joint linkage association mapping (JLAM), markers were further filtered to retain SNPs with <1% missing values. As a result, a set of 7,490 SNPs that are uniformly distributed across the genome were retained for JLAM analyses. BLUPs across locations were used for the analyses. A linear model comprising co-factors and population effect (Würschum, 2012) was used to implement JLAM as it performs well for association analysis in segregating populations. This model was explained in detail by Liu et al. (2011) and Würschum (2012). In brief, with this model, in a first step, stepwise multiple linear regression was used in addition to population effect to select the cofactors based on the Schwarz Bayesian criterion (Schwarz, 1978), and, in the second step, *P-*values for the association of each marker with phenotypic value were calculated for the *F*-test by comparing a full model (including SNP effect) against a reduced model (without SNP effect) [for details see Reif et al. (2010)]. R software version 4.2.1 (R_Core_Team, 2023) was used to carry out genome-wide scans for QTLs, and cofactors were selected using PROC GLM SELECT from SAS 9.4 (SAS Institute, 2015).

### Genome-wide association analyses

The raw GBS data set was subjected to a filter at 90% minimum count, with a MAF > 0.05 and heterozygosity < 5% to retain high-density markers. After these quality checks, 337,110 high-quality SNPs were retained for GWAS analyses. BLUPs across locations were used as phenotypes in association mapping scans. Population structure and linkage disequilibrium plots were already reported in earlier studies (Kibe et al., 2020b), so we used the same information in this study. Principal components (PC) were calculated using TASSEL version 5.2 (Bradbury et al., 2007). The R package “FarmCPU-Fixed and random model Circulating Probability Unification” with GAPIT (Genome Association and Prediction Integrated Tool) was used for GWAS analysis (Tang et al., 2016). The first three PCs were used in the model. A false discovery rate (FDR; p < 0.05) was used to correct for multiple testing while determining the significance threshold. To summarize GWAS results per chromosome, Manhattan scatter plots were generated. The −log10 *P-*values for all the analyzed SNPs for NCLB disease severity data were used to construct the Manhattan plots. Quantile-quantile (Q-Q) plots were plotted from the estimated −log10 (P) from the association panel for the NCLB disease severity trait.

SNPs detected in the association panel or from JLAM results were examined as polymorphisms in linkage disequilibrium with putative candidate genes from the “B73” RefGen_v2. Candidate genes were identified through BLAST searches against the “B73” RefGen_v2. (https://www.maizegdb.org/gbrowse/maize_v2). Putative candidate genes were selected by delving into the information gene ontology, Kyoto Encyclopedia of Genes and Genomes, and protein families (Pfam) (Ashburner et al., 2000; Bateman et al., 2004; Kanehisa and Goto, 2000). The presence of the protein-coding genes was searched within the range of 10 kb (5 kb upstream and downstream) in the vicinity of the detected SNPs.

### Genomic prediction

BLUEs across environments for the IMAS panel, each of the DH populations, and across DH populations were used for the analysis. Because all DH populations and IMAS panel were genotyped with GBS SNPs, we filtered in single file by excluding SNPs with MAF < 5%, heterozygosity > 5%, and no missing values. For all populations, the same set of high-quality uniformly distributed 4,000 SNPs was used. Among different GS models, ridge regression BLUP (RR-BLUP; Meuwissen et al., 2001; Endelman, 2011) appears well suited for regular plant breeding trials (Albrecht et al., 2011). Moreover, RR-BLUP has the advantage of being computationally less intensive, which is important in cross-validation studies. Therefore, we used RR-BLUP in the present study. GP analyses were conducted by using the package rrBLUP in R program (Endelman, 2011; Zhao et al., 2012; Crossa et al., 2017; R_Core_Team, 2023). We applied five-fold cross-validations in three different scenarios: the “within population” approach where both training and estimation set are derived from within each DH population and IMAS panel called as within-within approach. The second scenario is across-within populations, where all DH populations together form training set and individual DH population will be an estimation set. A third scenario is a combined population prediction approach where either all DH populations were combined and/or all DH populations together with IMAS panel were used to form both training and estimation sets. For each scenario, 100 iterations were performed for the sampling of the training and estimation sets. The predictive ability within each scenario (within-within, across-within, and combined all) was estimated by the Pearson correlation coefficient between the corrected phenotypic values (BLUEs) for each population and their predicted Genomic estimated breeding values (GEBVs).

The genotype-by-environment interaction plays a key role in genome selection and prediction in multi-environmental trials, across environments or at each environment usually is improved modeling the genotype-by-environment interaction (Burgueño et al., 2008). We evaluated three different models using the IMAS panel to evaluate prediction accuracy by doing prediction by environment or considering the genotype-by-environment interaction. The first model is a simple model in which each environment is analyzed individually. The second model includes an environmental effect in it, but marker effects are the same across environments. Finally, we fit a model in which marker effects vary by environment, it is a marker by environment interaction component. The model includes an overall marker effect across environments plus a specific environmental effect of each marker (Lopez-Cruz et al., 2015). For the analysis, we used a modified version of the scripts presented in github.com/MarcooLopez/Genomic-Selection/blob/master/multi_environment.mdfollowed the scripts. Here, we mimic two scenarios of prediction: one in which 82 genotypes (30%) were not evaluated in any environment (CV1) and the second in which 82 (30%) genotypes were not tested in two of six environments (CV2). Prediction accuracy was measured as the Pearson correlation between observed and predicted values in each environment and across environments.

## Results

### Phenotypic evaluation resistance to northern corn leaf blight in tropical maize germplasm

One association panel, three DH, and three F_3_ populations were evaluated in NCLB disease hotspots in Kenya. Assessments of NCLB disease severity for each population at each location revealed significant genotypic variances (**Supplementary Table S1**), indicating sufficient disease pressure and differential response among the tested genotypes. In addition to genotypic variations, there were significant positive correlations between locations for each population (**Supplementary Table S2**). The locations selected for assessing each population differed in terms of NCLB disease severity (significant environmental variations). These variations offered valuable insights into the reaction of individual lines to NCLB at different locations.

**Figure 1:**
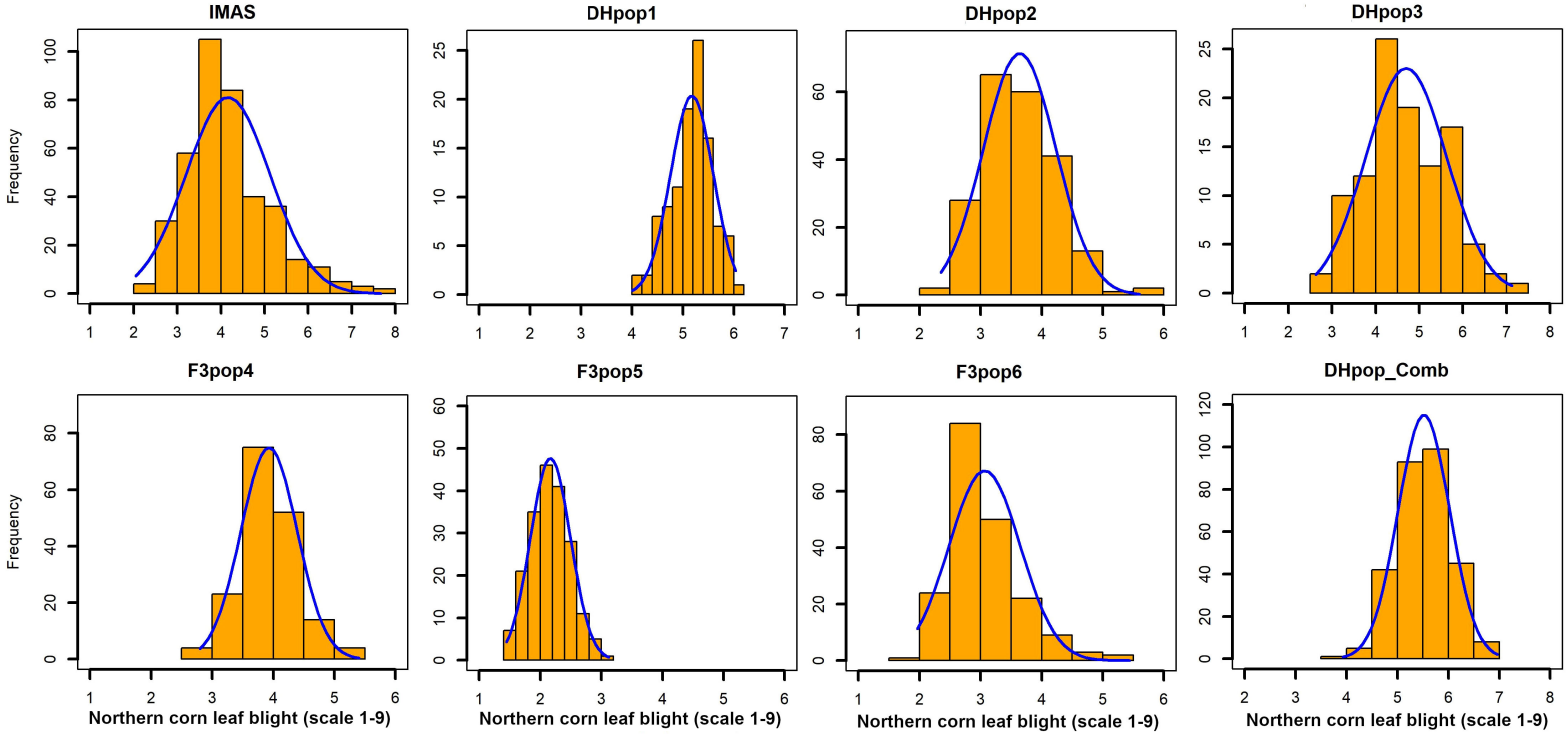
Phenotypic distribution of NCLB disease severity scores (on a 1-to-9 scale) in IMAS association panel, three DH populations, and combined DH populations, and three F_3_ populations evaluated across locations.

**Figure 2:**
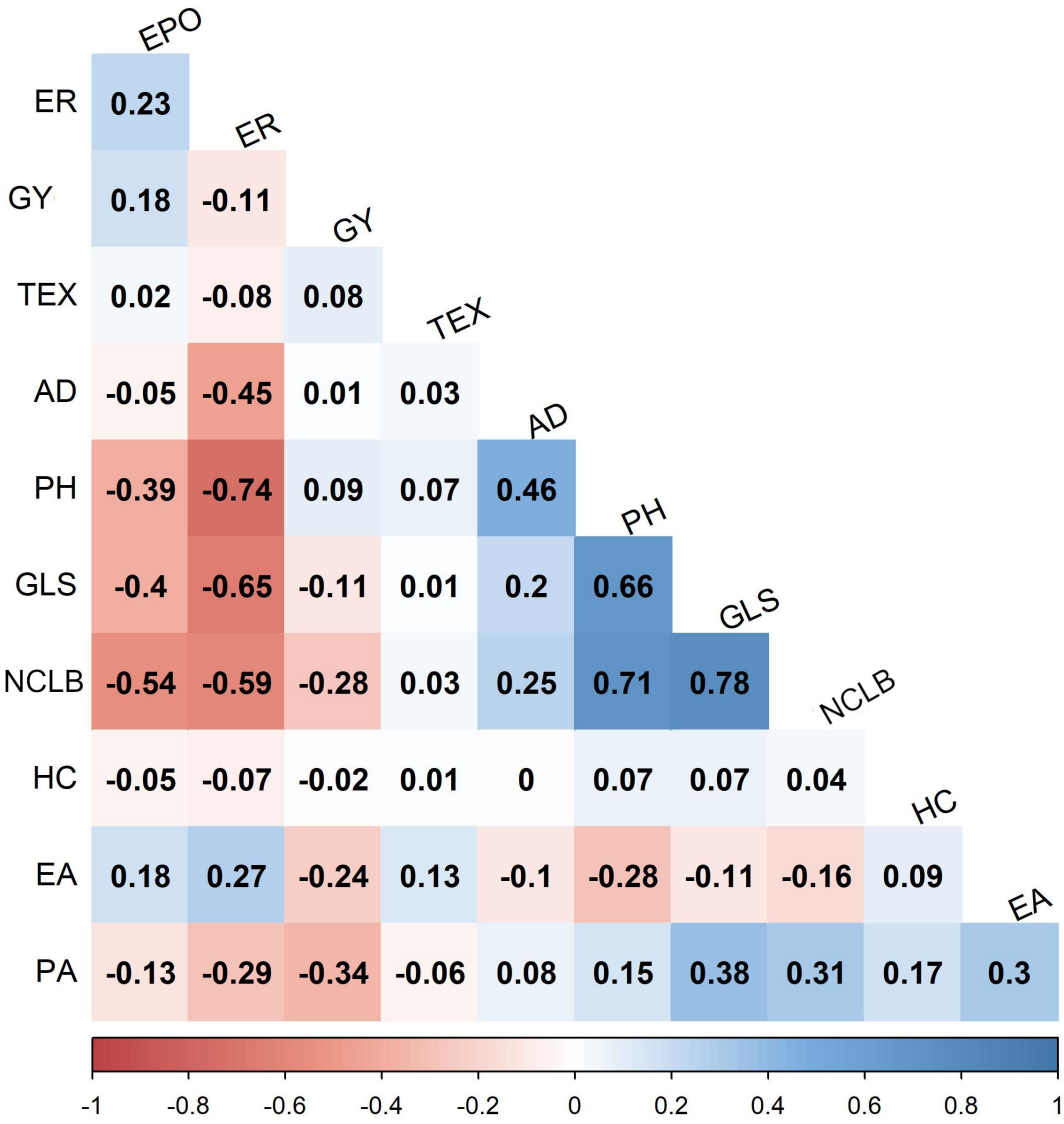
Pairwise phenotypic correlations between northern corn leaf blight scores and other traits in combined three DH populations evaluated in three environments. Correlation values > 0.10 and >0.15 were interpreted as significant at 0.05 and 0.01 levels, respectively. GYG, grain yield; AD, days to anthesis; PH, plant height; EH, ear height; EPO, ear position; HC, husk cover; GLS, gray leaf spot; NCLB, Northern corn leaf blight; ER, ear rot; EA, ear aspect; PA, plant aspect.

Analyses of variance within populations and across environments revealed significant variations in disease severity (**Table 1**). On a scale of 1.0–9.0, NCLB disease severity was high in DH populations, with mean scores of 5.17 (CML494 × CML550 DH pop 1) and 4.69 (CML511 × CML550 DH pop 3). Across the studied populations, CZL0723 × CZL0719 F_3_ pop 6 (mean = 3.06) and CZL0009 × CML505 F_3_ pop 5 (mean = 2.21) had the lowest disease severity scores (**Table 1**). The distribution frequency of mean NCLB disease severity scores resembled a normal pattern (**Figure 1**). Across environments, analysis of variance revealed significant genotypic and G × E variations (*P* < 0.05) in all populations except for CZL0723 × CZL0719 F_3_ pop 6 (**Table 1**). Broad-sense heritability was moderate to high. In the correlation analysis, NCLB disease severity was positively and significantly correlated to AD, PH, and GLS (**Figure 2**). However, NCLB was negatively and significantly correlated to GY, ER, EPO, and EA. GY was positively and significantly correlated with PH and EPO but negatively correlated with ER, GLS, EA, and PA. ER was negatively and significantly correlated with NCLB, GLS, PH, AD, and GY (**Figure 2**). PH was positively correlated with AD and GLS.

### QTLs associated with resistance to NCLB in tropical maize populations

The maize populations used in this study for QTL mapping were also evaluated in earlier studies (Ertiro et al., 2020a; Kibe et al., 2020a; Sitonik et al., 2019), which provided detailed genetic map information. **Table 2** shows details of the QTLs associated with NCLB in the studied tropical maize populations. Three QTLs associated with NCLB were detected in DH pop 1, which explained a total phenotypic variation of 34.3% (**Table 2**). The detected QTLs were found on chromosomes 2 and 7. For DH pop 2, six QTLs were detected on chromosomes 1, 2, 6, 9, and 10. These QTLs together explained 51.4% of total phenotypic variation. In DH pop 3, four QTLs were detected; among them, two QTLs were located on chromosome 3 and the others on chromosomes 1 and 10. Together, they explained 41.1% of the total phenotypic variation. In F_3_ pop 4, two QTLs were identified which together explained 12.5% of total PVE. In F_3_ pop 5, four QTLs were detected for resistance to NCLB, which together explained 12.7% of the total PVE. In F_3_ pop 6, there were four QTLs detected which together explained 14.7% of the total phenotypic variations. The proportion of PVE explained by each QTL in each population varied from 10.2% to 15.8% for DH pop 1, 2.8% to 15.8% for DH pop 2, 9.9% to 13.1% for DH pop 3, 5.9% and 9.6% for F_3_ pop 4, 6.2% to 9% for F_3_ pop 5, and 4.1% to 10.9% for F_3_ pop 6 (**Table 2**).

**Table 2:** Analysis of QTLs associated with NCLB resistance in DH and F_3_ biparental populations evaluated in multiple environments.

Trait	Chr	Position (cM)	LOD	PVE (%)	Add	Dom	TPVE (%)	Flanking markers		QTL name
CML550 × CML494 (DH pop 1)										
NCLB	2	2	6.06	15.77	0.04	–	34.28	*S2_197381415*	*S2_197936812*	*qNCLB2-197*
	2	319	4.66	11.75	−0.04	–		*S2_15120146*	*S2_14108062*	*qNCLB2-14*
	7	124	4.06	10.16	0.04	–		*S7_17180908*	*S7_17479195*	*qNCLB7-17*
CML550 × CML504 (DH pop 2)										
NCLB	2	172	9.13	6.86	0.06	–	51.37	*S2_200406183*	*S2_199173754*	*qNCLB2-199*
	4	67	18.70	15.81	−0.10	–		*S4_217096503*	*S4_216318317*	*qNCLB4-216*
	5	474	4.59	3.30	0.04	–		*S5_27062274*	*S5_24712589*	*qNCLB5-24*
	6	232	3.82	2.76	−0.04	–		*S6_159006932*	*S6_161010798*	*qNCLB6-161*
	7	220	7.66	5.80	0.06	–		*S7_127579232*	*S7_126523093*	*qNCLB7-126*
	8	280	12.15	9.62	0.07	–		*S8_146976545*	*S8_146475249*	*qNCLB8-146*
CML550 × CML511 (DH pop 3)										
NCLB	1	323	3.34	10.40	−0.11	–	41.12	*S1_244433411*	*S1_238464323*	*qNCLB1-238*
	3	40	4.07	13.05	−0.12	–		*S3_214123437*	*S3_213338610*	*qNCLB3-213*
	3	367	3.22	9.89	0.10	–		*S3_45037474*	*S3_40065285*	*qNCLB3-40*
	10	173	3.70	11.80	0.12	–		*S10_90044674*	*S10_84229674*	*qNCLB10-84*
CZL074 × LaPostaSeqC7-F103-1-2-1-1 (F_3_ pop 4)										
NCLB	1	284	3.53	5.88	−0.17	-0.08	12.46	*S1_46406150*	*S1_33884416*	*qNCLB1-40*
	8	108	3.01	9.60	−0.26	0.01		*S8_27220841*	*S8_64174106*	*qNCLB8-30*
CZL00009 × CML505 (F_3_ pop 5)										
NCLB	4	572	6.02	6.80	0.24	0.03	12.1	*S4_204127436*	*S4_222798122*	*qNCLB4-220*
	4	581	8.59	8.98	−0.29	0.05		*S4_222798122*	*S4_224911596*	*qNCLB4-222*
	8	217	5.11	6.25	−0.22	0.04		*S8_21874363*	*S8_19295622*	*qNCLB8-20*
	8	224	7.61	7.96	0.26	0.02		*S8_19295622*	*S8_14702663*	*qNCLB8-19*
CZL0723 × CZL0719 (F_3_ pop 6)										
NCLB	3	221	3.69	4.10	0.16	−0.27	14.66	*S3_54472637*	*S3_34440945*	*qNCLB3-50*
	4	279	3.23	10.95	−0.43	−0.03		*S4_237390755*	*S4_236648145*	*qNCLB4-237*
	8	80	4.42	5.47	−0.31	−0.12		*S8_147263937*	*S8_135592710*	*qNCLB8-146*
	8	83	3.49	5.55	0.32	−0.13		*S8_135592710*	*S8_134080585*	*qNCLB8-135*

Chr, chromosome; LOD, logarithm of odds; cM, centiMorgan units; add, additive effect; TPVE, total phenotypic variance explained; QTL name composed by the trait code followed by the chromosome number in which the QTL was mapped and a physical position of the QTL; NCLB, northern corn leaf blight.

Through JLAM, we identified 37 QTLs associated with resistance to NCLB. These QTLs were distributed in all 10 chromosomes and together explained 49.4% of the total PVE (**Table 3**). Unlike linkage mapping where phenotypic variation explained by each QTL showed minor to major effects (2.8%–15.8%), JLAM-detected QTLs showed minor effects, ranging from 0.5% to 2.1%. Most of the QTLs identified through JLAM were located on chromosome 2 (eight QTLs) followed by chromosome 5 (seven QTLs). Large variation was observed in SNP allele substitution effects which varied between −0.48 and 0.34 (**Table 3**). The putative candidate genes associated with these identified QTLs and their predicted function are also reported in **Table 3**, which are either directly or indirectly involved in plant defense (**Table 3**).

**Table 3:** Analysis of NCLB-associated markers, allele substitution (α) effects, and the explained proportion of phenotypic variance (R^2^) of the joint linkage association mapping in multiple segregating DH populations.

SNP^a^	QTL name	Chr	α-Effect	PVE (%)	Putative candidate	Predicted function of candidate gene
*S1_3010423*	*qNCLB1-3*	1	−0.07	0.70	*GRMZM2G137236*	Cell vesicle transport Adaptor protein complex AP-2, alpha subunit
*S1_36801883*	*qNCLB1-36*	1	−0.06	0.50	*GRMZM2G150827*	Dynamin-related protein 4C (DRP4C)
*S1_248124348*	*qNCLB1-248*	1	0.13	0.80	*GRMZM2G110304*	RNA regulation of transcription putative transcription regulator
*S1_287290870*	*qNCLB1-287*	1	−0.10	1.70	*GRMZM2G178415*	N-metabolism N-degradation, glutamate dehydrogenase
*S2_4464978*	*qNCLB2-4*	2	0.06	0.50	*GRMZM2G076212*	Serine/threonine-protein kinase SD2-5
*S2_7207049*	*qNCLB2-7*	2	0.16	0.70	*GRMZM2G419290*	Putative G-type lectin S-receptor-like serine/threonine-protein kinase
*S2_10474509*	*qNCLB2-10*	2	−0.20	1.10	*GRMZM2G044629*	UDP-N-acetylglucosamine diphosphorylase 2/cell wall precursor synthesis
*S2_15475173*	*qNCLB2-15*	2	0.14	1.50	*GRMZM2G493586*	Uncharacterized
*S2_136562142*	*qNCLB2-136*	2	0.11	0.50	*GRMZM2G003234*	RNA regulation of transcription. C2H2 zinc finger family C2H2-like zinc finger protein
*S2_148660794*	*qNCLB2-148*	2	−0.18	1.20	*GRMZM2G115658*	ABC transporters and multidrug resistance systems zinc-induced facilitator
*S2_186533942*	*qNCLB2-186*	2	0.23	2.00	*GRMZM2G123972*	Uncharacterized
*S2_197936812*	*qNCLB2-197*	2	−0.11	2.10	*GRMZM2G021587*	RNA regulation of transcription
*S3_114875183*	*qNCLB3-114*	3	−0.48	1.80	*GRMZM2G176282*	EXS, C-terminal
*S3_123784490*	*qNCLB3-123*	3	0.34	0.90	*GRMZM2G173280*	RNA regulation of transcription unclassified Remorin family protein
*S3_200187150*	*qNCLB3-200*	3	−0.06	0.40	*GRMZM2G078756*	Phenylalanine-tRNA ligase phenylalanyl-tRNA synthetase class IIc family protein
*S3_209548358*	*qNCLB3-209*	3	0.13	0.50	*GRMZM2G078926*	Signaling, leucine-rich repeat protein kinase family protein
*S3_212501774*	*qNCLB3-212*	3	0.05	0.30	*GRMZM2G160971*	Uncharacterized
*S4_157115413*	*qNCLB4-157*	4	−0.07	0.30	*GRMZM2G570925*	Uncharacterized
*S4_218847954*	*qNCLB4-218*	4	−0.10	1.7	*GRMZM2G057402*	C2 domain–containing protein calcium-dependent lipid-binding family protein
*S5_5924352*	*qNCLB5-5*	5	−0.12	0.90	*GRMZM2G114557*	Protein degradation Peptidase S24/S26A/S26B/S26C family protein
*S5_8350170*	*qNCLB5-8*	5	−0.09	0.20	*GRMZM2G100380*	Cell organization ankyrin repeat family protein/ankyrin repeat-containing protein At5g02620
*S5_8409630*	*qNCLB5-8*	5	0.18	0.80	*GRMZM2G065669*	Unknown
*S5_26100208*	*qNCLB5-26*	5	−0.21	0.30	*GRMZM2G022175*	Putative RING zinc finger domain superfamily protein
*S5_183126431*	*qNCLB5-183*	5	0.09	0.70	*GRMZM2G007466*	postranslational modification Integrin-linked protein Kinase family
*S5_209733109*	*qNCLB5-209*	5	−0.14	0.30	*GRMZM2G163776*	Uncharacterized
*S5_215991184*	*qNCLB5-215*	5	−0.06	0.80	*GRMZM2G064603*	ABC transporter G family member 28/transport ABC transporters and multidrug resistance systems
*S6_165014618*	*qNCLB6-165*	6	0.07	0.90	*GRMZM2G065757*	Protein degradation aspartate protease
*S7_127888863*	*qNCLB7-127*	7	−0.09	1.80	*GRMZM2G140633*	Cell cycle, encodes a cyclin involved in cell proliferation during stomatal cell lineage development
*S8_21847291*	*qNCLB8-21*	8	0.11	0.30	*GRMZM2G032551*	Uncharacterized
*S8_35814899*	*qNCLB8-35*	8	−0.18	0.90	*GRMZM2G001024*	Uncharacterized
*S8_146017787*	*qNCLB8-146*	8	−0.08	0.70	*GRMZM2G017523*	Uncharacterized
*S8_164859738*	*qNCLB8-164*	8	0.15	1.00	*GRMZM2G050693*	Uncharacterized
*S8_170127444*	*qNCLB8-170*	8	−0.08	0.90	*GRMZM2G036448*	Transmembrane amino acid transporter family protein
*S9_8467793*	*qNCLB9-8*	9	−0.17	1.70	*GRMZM2G066373*	Uncharacterized
*S9_140347342*	*qNCLB9-140*	9	−0.06	0.30	*GRMZM2G179329*	Uncharacterized
*S9_153223374*	*qNCLB9-153*	9	0.09	0.90	*GRMZM2G442769*	Uncharacterized
*S10_12104511*	*qNCLB10-12*	10	−0.11	0.50	*GRMZM2G004060*	WRKY transcription factor 15 (wrky15)
**Total PVE (%)**				**49.4**		

Chr, chromosome; PVE, proportion of phenotypic variance explained; NCLB, northern corn leaf blight; ^a^the exact physical position of the SNP can be inferred from the marker’s name, for example, *S1_82702920*: chromosome 1; 82,702,920 bp.

**Figure 3:**
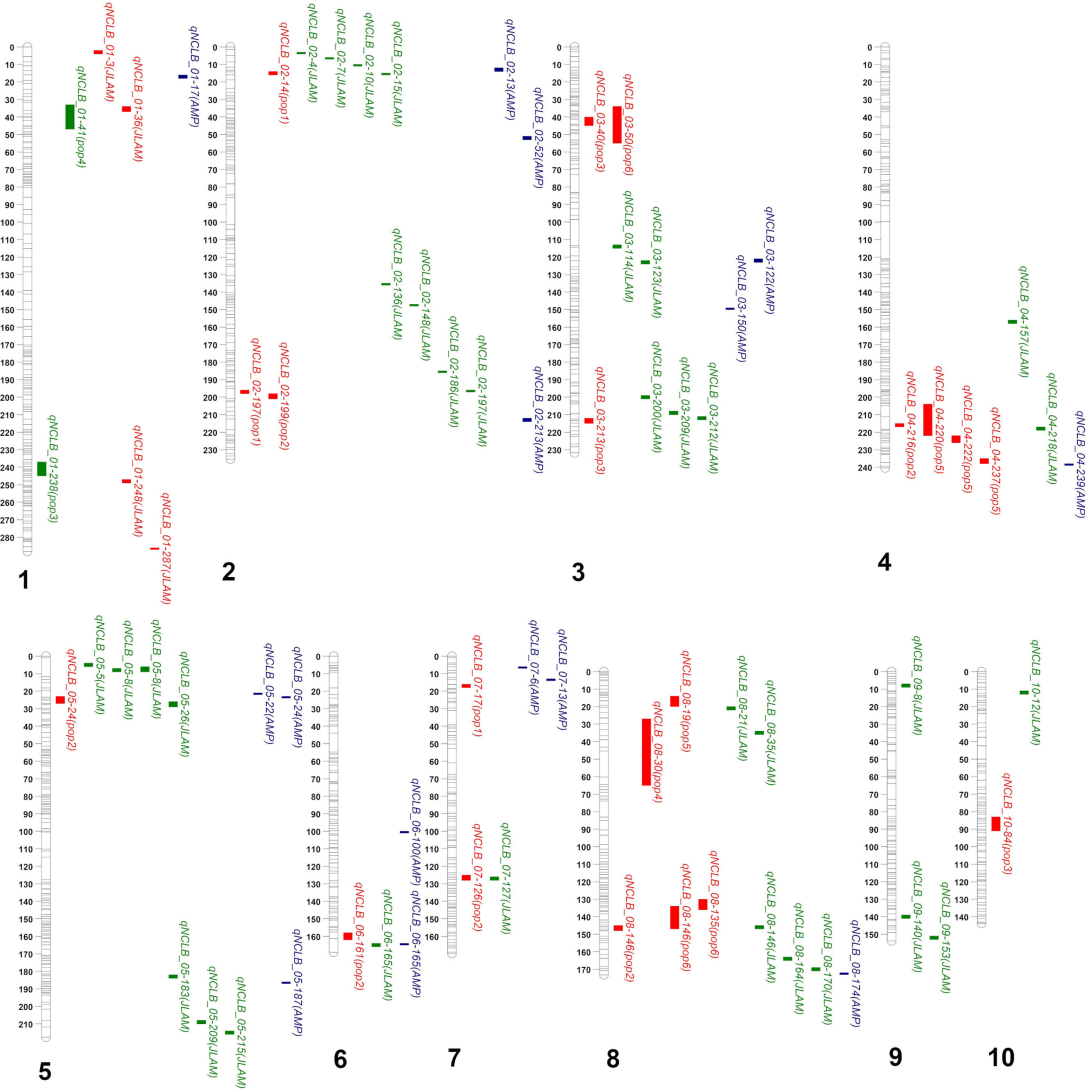
Mapping of NCLB resistance-associated QTLs detected based on individual biparental-based linkage mapping, joint linkage association mapping (JLAM), and IMAS association mapping panel (AMP). Red, green, and blue colors represent QTL detected under biparental-based linkage mapping (pop 1, pop 2, pop 3, pop 4, pop 5, and pop 6), JLAM, and AMP approaches, respectively.

The QTLs or SNPs detected through GWAS, JLAM, and linkage mapping were compared on a physical map, which revealed several QTLs overlapped at same genomic regions (**Figure 3**). The SNP detected through JLAM at 36 Mbp (*qNCLB1_36*) is located within the QTL region detected in F3 pop4 (*qNCLB-01-41*). QTL detected in DH pop3 (*qNCLB1_238*) is adjacently located with SNP detected through JLAM (*qNCLB1_248*). Among the 37 QTLs identified through JLAM, 10 were co-located within the QTLs identified through individual population-based QTL mapping (**Tables 2**, **3**). On chromosome 1, *SNP S1_36801883* was co-located within the QTL *qNCLB1-40* identified in F_3_ pop 6 (**Table 2**). On chromosome 2, SNP *S2_15475173* and *S2_197936812* were co-located within the QTL *qNCLB2-14* and *qNCLB2-197*, respectively (identified in DH pop 1). SNP *S4_218847954* was co-located within the QTL (*qNCLB4-220*) from F_3_ pop 5. SNPs *S5_26100208* and *S7_127888863* were co-located within the QTLs detected in DH pop 2, *qNCLB5-24* and *qNCLB7-126* on chromosomes 5 and 7, respectively. Three SNPs from chromosome 8—*S8_21847291*, *S8_35814899*, and *S8_146017787*—were co-located within the QTLs *qNCLB8-20* from pop 5, *qNCLB8-30* from pop 4, and *qNCLB8-146* from pop 6, respectively (**Tables 2**, **3**).

### Genome-wide association study for NCLB resistance in tropical maize germplasm

**Figure 4:**
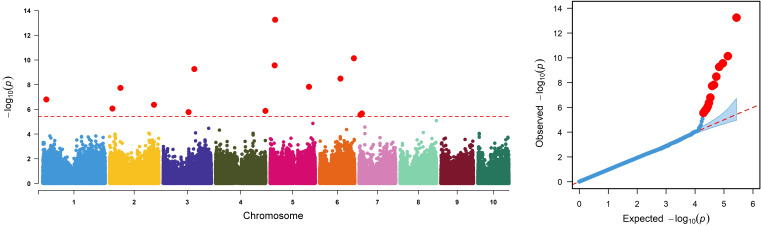
Manhattan and quantile-quantile (Q-Q) plots generated using a mixed linear model for NCLB scores across environments. The x-axis indicates the SNP location along the 10 chromosomes, with chromosomes separated by different colors. The significance level was set at P = 2 × 10^–5^ at 0.05 false discovery rate (FDR) and is represented on the plot by the dashed horizontal line. The position of SNPs along the 10 maize chromosomes is shown on the x-axis, with each color indicating distinct maize chromosomes. The −log10(P observed) is shown on the y-axis.

The IMAS panel was used to identify and validate genomic regions for resistance to NCLB through GWAS. Fifteen marker–trait associations were identified by using 337,110 high-quality SNPs filtered from the raw GBS data set (**Table 4**; **Figure 4**). Because both fixed and random effect models are iteratively used in FarmCPU, which helps in avoiding overfitting of the model by stepwise regression (Liu et al., 2016), we used the FarmCPU model in the association mapping. The detected markers were distributed across all chromosomes except chromosomes 9 and 10, with P-values ranging from 5.58 × 10^−14^ to 8.29 × 10^−06^. The Manhattan plot revealed the highest peak on chromosome 5 (*S5_24157791*). Our analysis identified SNPs whose physical coordinates co-localized with chromosome bins where QTLs associated with NCLB resistance had been previously reported. For instance, SNP *S6_165023408* was co-located with QTL *qNCLB6-165* [detected in JLAM (**Tables 3**, **4**)]. Using B73 maize genome V2.0, predicted gene annotations were studied to identify putative candidate genes associated with NCLB resistance. Numerous SNP associations identified in this study were situated within genes featuring functional domains related to metabolism, stress tolerance, and plant development. For example, SNP *S2_213818302* was associated with peroxidase activity and oxidase stress responses. SNP *S6_100083188*, on the other hand, was associated with the gene responsible for phosphoglycerate kinase (PGK) activity that is involved in plant defense response (**Table 4**).

**Table 4:** Highly significant SNPs identified in GWAS analysis of DH and IMAS panel that were evaluated for NCLB resistance.

SNP name^a^	Chr	MLM P-value	MAF	FDR P-values	SNP effect	Putative candidate genes	Predicted function of candidate gene
*S1_17844737*	1	1.60E-07	0.29	0.01	−0.10	*GRMZM2G059020*	ATP-dependent helicase activity
*S2_13777598*	2	8.71E-07	0.14	0.02	−0.11	*GRMZM2G031981*	HSP binding, protein folding
*S2_52683494*	2	1.87E-08	0.21	0.00	0.12	*GRMZM2G101730*	Unknown
*S2_213818302*	2	4.34E-07	0.24	0.01	−0.10	*GRMZM2G450717*	Peroxidase activity, heme binding, oxidative stress response
*S3_122496339*	3	1.69E-06	0.18	0.04	0.10	*GRMZM2G340251*	ATP binding
*S3_150390803*	3	5.44E-10	0.27	0.00	0.11	*GRMZM2G176968*	Quercetin sulphate biosynthesis
*S4_239705109*	4	1.34E-06	0.43	0.03	−0.08	*GRMZM2G150337*	G protein–coupled receptor signaling pathway
*S5_22035704*	5	2.78E-10	0.10	0.00	0.17	*GRMZM2G549959*	Unknown
*S5_24157791*	5	5.58E-14	0.29	0.00	0.16	*GRMZM2G107444*	Response to freezing
*S5_187085620*	5	1.49E-08	0.42	0.00	−0.10	*GRMZM2G042173*	Protein binding
*S6_100083188*	6	3.25E-09	0.49	0.00	0.10	*GRMZM5G811022*	Phosphoglycerate kinase activity
*S6_165023408*	6	7.32E-11	0.11	0.00	−0.19	*GRMZM2G366795*	WRKY transcription factor 51
*S7_6577768*	7	2.79E-06	0.22	0.05	−0.09	*GRMZM2G032266*	Unknown
*S7_13616198*	7	2.18E-06	0.28	0.05	0.08	*GRMZM2G458494*	Uncharacterized
*S8_174614781*	8	8.29E-06	0.42	0.15	−0.07	*GRMZM2G118770*	Oxidoreductase, malic enzyme activity

MAF, minor allele frequency; ^a^the exact physical position of the SNP can be inferred from SNP’s name, for example, *S1_82702920*: chromosome 1; 82,702,920 bp.

### Genome-wide prediction accuracies of NCLB resistance in tropical maize populations

**Figure 5:**
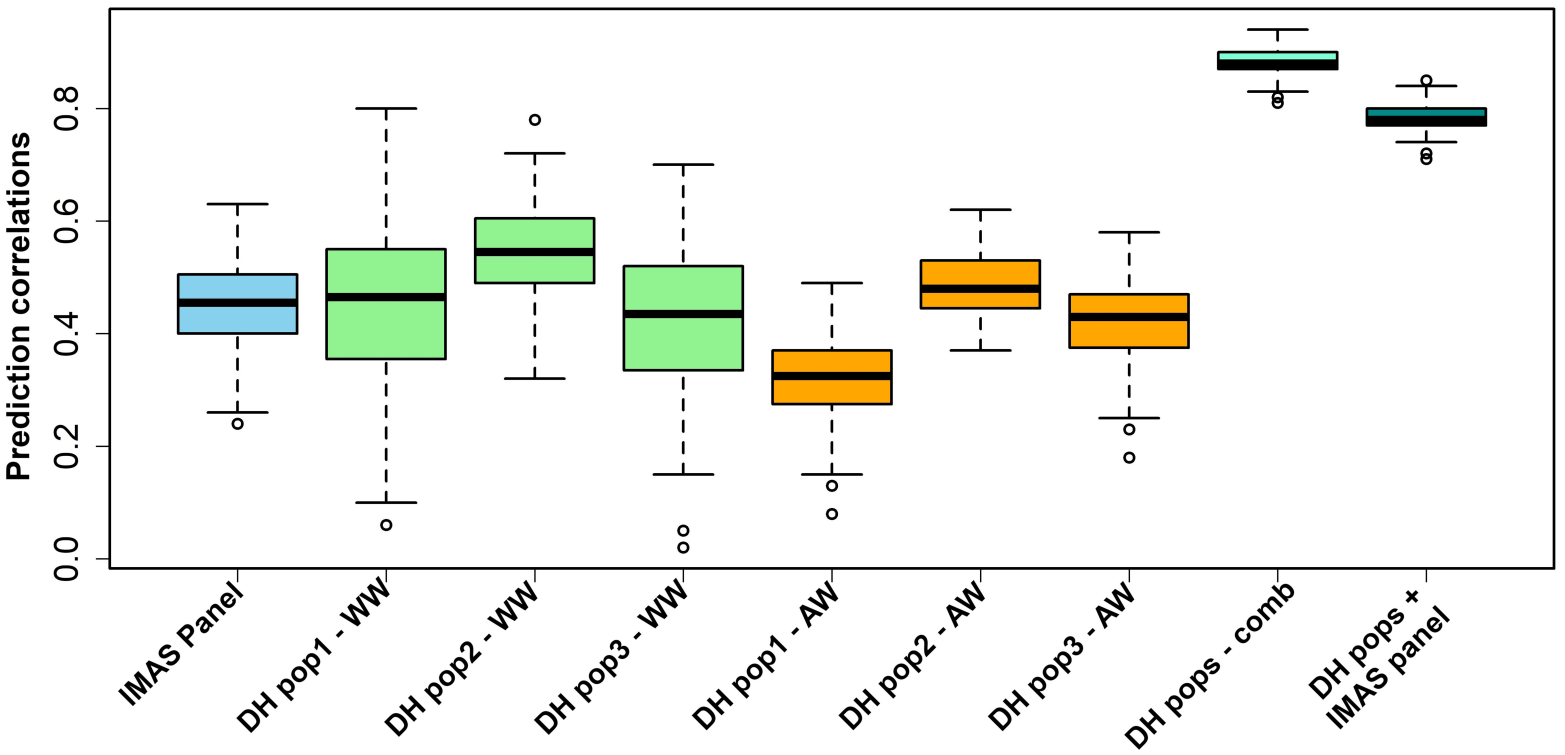
Genome-wide prediction correlations for NCLB resistance in biparental and IMAS association panel based on three different scenarios. WW (within-within) scenario, estimation and prediction sets are derived within populations; AW (across-within) scenario, combined populations serve as a training set and an estimation set is from single biparental population; and combined scenario, where combine all populations and randomly derive both training and testing set and evaluate with five-fold cross-validation.

RR-BLUP has the advantage of being computationally less intensive, which is important in cross-validation studies and suits well for routine application in plant breeding trials (Albrecht et al., 2011). Therefore, we used the RR-BLUP model (Endelman, 2011) to estimate the performance of tropical maize genotypes under NCLB disease pressure (**Figure 5**). The prediction accuracy was highest for combined all three DH populations (r = 0.88) followed by prediction across DH populations and GWAS panel (r = 0.79). Overall, the prediction accuracies across genotypes were moderate to high (0.32 to 0.88). Average prediction accuracies within DH populations were relatively higher: 0.44, 0.55, and 0.42 for DH pop 1, DH pop 2, and DH pop 3, respectively. The IMAS association panel had a prediction accuracy of 0.45. For the across-within prediction scenario, the accuracy values were 0.32, 0.49, and 0.42 when estimation set is DH pop 1, DH pop 2, and DH pop 3, respectively.

**Table 5** shows the results of genome-wide prediction correlations using and not using marker by environment effect or, similarly, genotype-by-environment effect. The major difference between by environment and across environment prediction is because, for by environment prediction, we used un-tested genotypes to calculate Pearson correlation, whereas, for across environment prediction, we used all genotypes, because all genotypes were missing in at least two environments. As usual, under CV2 cross-validation, the prediction accuracy is high because there are not completely untested genotypes. The comparison between models shows that the across model is better in average to predict by environments in four and five environments in CV1 and CV2, respectively. The single model without marker by environment interaction was only better than the other models in CV1 to predict at location Embu12. The model with marker by environment interaction presented the largest prediction accuracy in one environment in each, CV1 (Alupe14) and CV2 (Embu12), and to do prediction across environments.

**Table 5:** Average Pearson correlation and standard deviation (n = 100), between observed and predicted NCLB disease severity values by environment and across environment in two cross-validation scenarios.

Environment	CV1			CV2		
	Single	Across	M × E	Single	Across	M × E
Alupe11	0.352 (0.084)	0.410 (0.088)	0.384 (0.085)	0.350 (0.073)	0.712 (0.042)	0.677 (0.047)
Alupe12	0.329 (0.092)	0.384 (0.095)	0.354 (0.091)	0.329 (0.088)	0.680 (0.059)	0.663 (0.056)
Alupe13	0.358 (0.075)	0.404 (0.089)	0.385 (0.079)	0.365 (0.084)	0.712 (0.048)	0.694 (0.049)
Embu12	0.327 (0.082)	0.266 (0.088)	0.312 (0.083)	0.332 (0.080)	0.445 (0.066)	0.475 (0.064)
Kibos11	0.259 (0.096)	0.332 (0.093)	0.325 (0.095)	0.253 (0.086)	0.676 (0.065)	0.651 (0.061)
Alupe14	0.400 (0.091)	0.403 (0.081)	0.408 (0.090)	0.393 (0.092)	0.695 (0.063)	0.649 (0.068)
Across environment, all genotypes	0.856 (0.021)	0.875 (0.021)	0.878 (0.021)	0.913 (0.006)	0.963 (0.004)	0.967 (0.003)

CV1 and CV2, using three different models, individual environment analysis (single), across environment without marker by environment interaction (across), and across environment with marker by environment interaction (M × E).

## Discussion

### Phenotypic evaluation of tropical maize populations in NCLB hotspots

Assessing the genetic architecture of complex disease-resistance traits in plants requires extensive and accurate phenotyping. In environments where pathogen genetic diversity and disease pressure are high, broad-based quantitative resistance is essential because it is less broken by pathogen evolution (Galiano-Carneiro and Miedaner, 2017). Quantitative resistance to NCLB is governed by many genes (polygenic), with most QTL having minor effects and few having major phenotypic effects (Poland et al., 2011; Van Inghelandt et al., 2012). In this study, the NCLB scores for the IMAS association panel and each biparental population showed that resistance to NCLB in tropical maize is quantitative (**Figure 1**). This was well supported by earlier studies on association panels (Poland et al., 2011; Van Inghelandt et al., 2012) and biparental maize populations (Ranganatha et al., 2021; Omondi et al., 2023). We also observed significant genotypic and G × E interaction variances (**Table 1**). Estimates of broad sense heritability on a mean line basis (within individual populations) ranged from moderate to high. This suggests that the estimated variance within each population is reliable and indicates the potential for significant progress in selecting for NCLB resistance in this tropical maize germplasm.

Understanding the correlation and interactions between different traits is crucial in the development of marker-assisted-based multi-trait strategies (Xu and Crouch, 2008). Our results showed that NCLB has a negative and a significant relationship with grain yield. Similar findings of the negative relationship between NCLB and GY have been reported in earlier studies (Pataky et al., 1998; Weems, 2016; Razzaq et al., 2019). The negative correlation between plant height and NCLB in this study corroborated with Welz and Geiger (2000) who found a correlation coefficient of −0.27 (*P* = 0.01) between these two traits. The significant negative correlation indicates taller plants may have better resistance to NCLB. On the other hand, this will enhance lodging problems and having taller plants resistant to NCLB may not hold in different germplasm, so it warrants more studies before using in breeding selection.

### Linkage and joint linkage mapping and putative candidate genes

The SNP-based linkage maps are being used to map NCLB resistance via linkage mapping, helping researchers understand the function of the chromosomal region or loci at the gene level. In this study, we used three DH populations and three F_3_ populations and identified genomic locations on all chromosomes except on chromosomes 1 and 9 (**Table 2**). Six major effect QTLs (>10% PVE) were identified; among them, three were in DH pop1, located on chromosomes 2 and 7. The moderate effect QTLs on chromosome 2 (*qNCLB2_14* and *qNCLB2_197*) were detected within the QTL region reported in an earlier study that used DH populations (Omondi et al., 2023). The major effect QTL *qNCLB7_14* detected in DH pop 1 (bin 7.02) was also co-located by Wang et al. (2018) with 13.16% of phenotypic variance explained. Bin 4.09 is another important region that harbors NCLB resistance QTLs. Chromosomal bins 1.03–06, 4.04–06, 5.04–07, 8.02–03, 8.05–06, and 9.02–04 have been consistently identified across multiple QTL mapping studies (Rashid et al., 2020). Welz et al. (1999) also found five QTLs located on different chromosome bins 1.06/1.07, 3.07, 4.03, 5.04, and 6.05/6.06 in a temperate F_3_ population. Most of the QTLs detected in this study overlapped with earlier studies (Miedaner et al., 2020; Galiano-Carneiro et al., 2021; Ranganatha et al., 2021; Omondi et al., 2023), indicating the presence of consistent QTLs across regions. This supports the potential for enhancing NCLB resistance across different genetic backgrounds through either marker-assisted recurrent selection or genome-wide selection.

For JLAM, even though the total PVE explained by the 37 QTLs was 49.4%, individual phenotypic variation for each QTL was less than 2.5% (**Table 3**), indicating that NCLB resistance being a polygenic trait. As expected, JLAM improved the resolution within the QTL intervals, finding new QTLs that are hard to find in individual population-based linkage mapping (**Figure 3**). Six of the QTLs identified by JLAM lay within or just outside of the confidence intervals identified earlier (**Tables 2**, **3**; **Figure 3**). Many previous studies on resistance to NCLB have implicated the use of qualitative resistance in temperate and tropical germplasm (Welz and Geiger, 2000; Galiano-Carneiro and Miedaner, 2017). These are mostly *Ht* genes including *Ht1*, *Ht2*, *Ht3*, *Ht4*, *Htn1*, and *Html*, which could be partially or fully dominant (Carson, 1995; Welz and Geiger, 2000; Ogliari et al., 2005; Pataky and Ledencan, 2006). *HtP*, a dominant gene, and *rt*, a recessive gene conferring resistance to *E. turcicum* have been mapped on bins 2.08 and 3.06, respectively (Ogliari et al., 2007). These two regions were in proximity to SNPs *S2_197936812* (*qNCLB2-197*) and *S3_212501774* (*qNCLB3-212*) identified through JLAM (**Table 3**). Marker *S2_197936812* lies at the exact location of the upper confidence interval of QTL *qNCLB-197* identified through linkage mapping within DH pop 1, which coincidentally explained the highest phenotypic variation within that population. However, *S3_212501774* found on chromosome 3 fell outside the confidence interval of QTL *qNCLN3-213* in DH pop 3 (**Tables 2**, **3**). The QTL *qNCLB5-26* was co-located within the QTL *qNCLB5-24* identified in DH pop 2 at bin 5.03 (**Tables 2**, **3**). This is also a region identified for NCLB resistance through NAM in earlier studies (Poland et al., 2011). Interestingly, QTLs were identified in five genomic regions across chromosome 2 (10-15 Mbp), chromosome 4 (220-239 Mbp), chromosome 5 (22-26 Mbp), chromosome 6 (161-166 Mbp), and chromosome 8 (19-35 Mbp) using all three mapping approaches (**Figure 3**). These genomic regions where QTLs detected via linkage mapping, JLAM, and GWAS not only demonstrated stability but also contributed to narrowing down the confidence interval of the QTLs. These are the regions need to prioritize for future marker assisted breeding for NCLB resistance.

### Genome-wide association analyses

The identified significantly associated 15 SNPs were distributed on all maize chromosomes except chr 9 and 10. The total phenotypic variance explained by each SNP was <9%, indicating that resistance to NCLB is a polygenic in nature. Loci conferring resistance to NCLB has been detected through association mapping in previous studies (Wisser et al., 2006; Jamann et al., 2014). SNP *S8_174614781* identified in our GWAS lay within the upper confidence interval of QTL identified in linkage mapping within F_3_ pop 5. Likewise, *S6_165014618* on chromosome 6 identified via JLAM was closely associated with SNP *S6_165023408* identified via GWAS. Because we did not observe deviation of SNP toward the expected *P-*value of Q-Q plots, putative candidate genes associated with significant SNP gave greater confidence in response to NCLB disease severity (**Table 4**). SNPs *S1_17844737* on chromosome 1 and *S3_122496339* on chromosome 8 were associated with genes *GRMZM2G059020* and *GRMZM2G340251*, which code for ATP-dependent helicase activity and binding (**Table 4**). Using SNP-based bulk segregant analysis, Zhai et al. (2022) reported ATP-dependent helicase with disease resistance QTLs associated with NCLB in maize.

The candidate gene *GRMZM2G031981* associated with *S2_13777598* on chromosome 2 codes for heat shock protein (HSP) binding and folding. HSP proteins play a pivotal role within the intricate cellular system of molecular chaperones and catalysts for protein folding (Mayer and Bukau, 2005; Diogo-Jr et al., 2023). These HSPs function as chaperones by either enhancing the stability of newly synthesized proteins to facilitate proper folding or by aiding in the re-folding process of proteins that have been compromised due to cellular stress (Abou-Deif et al., 2019). HSPs are crucial for maintaining the integrity of PRRs on the cell membrane and R proteins inside the cell, ensuring that they are ready to counter potential threats (Park and Seo, 2015; Berka et al., 2022).

*GRMZM2G450717* on chromosome 2 encodes peroxidase activity. Peroxidase activity contributes to lignification in plant cell walls, facilitating the production of phenolic compounds and ultimately reinforcing cell walls to prevent pathogen intrusion during infection (Almagro et al., 2008; Yanti, 2015). Earlier studies have also documented the role that peroxidases in plant growth and development (Li, 2023). The gene was also associated with heme binding and oxidative response. Under stress, earlier studies have reported on the role of heme-mediated homeostasis in plants (Singh and Bhatla, 2022; Wu et al., 2022). Another SNP identified in this study, *S3_150390803*, was linked to *GRMZM2G176968*, which codes for quercetin sulphate biosynthesis. Under osmotic stress, quercetin has been shown to enhance seed germination and vigor (Yang et al., 2021). *GRMZM2G150337* gene that codes for G protein–coupled receptor signaling pathway was significantly associated with *SNP S4_239705109*. The G protein–coupled receptor genes have been shown in earlier studies to enhance chilling tolerance in maize (Zhou et al., 2023). Similarly, *GRMZM2G107444* that codes for response to freezing was associated with SNP *S5_24157791* on chromosome 5.

The candidate gene *GRMZM5G811022* associated with *S6_100083188* on chromosome 6 codes for PGK activity. In activating plant defense mechanisms, protein kinases have been shown to be important in signaling during pathogen recognition. In a GWAS for NCLB resistance study, Rashid et al. (2020) identified significant SNPs on chromosome 7 that were associated with a gene coding for the protein kinase superfamily.

*S3_150390803* and *S8_174614781* were linked to *GRMZM2G366795* and *GRMZM2G118770*, which codes for WRKY transcription factor 51, and oxidoreductase and malic enzyme activity, respectively. WRKY transcription factors, specific to plants, are significant regulators of the expression of genes involved in defense responses against pathogen attack (Huo et al., 2021). They also have been associated with regulating maize antioxidant defense under cadmium (Hong et al., 2017) and salt stress tolerance (Hu et al., 2021). On the other hand, malic enzymes (malate oxidoreductases), crucial in the photosynthetic C4 pathway (Ludwig, 2016; Bovdilova et al., 2019), facilitate the oxidative decarboxylation of malate into pyruvate, a reaction pivotal in several metabolic pathways. This study showed the usefulness of GWAS in revealing genomic regions associated with NCLB resistance. The identified genomic regions and candidate genes are crucial, and their validation would aid in comprehending the genetic and molecular mechanisms underlying NCLB host resistance.

### Prospects of genomic selection for northern corn leaf blight resistance in tropical maize

NCLB is known to carry both major effect (qualitative) and minor effect (quantitative) genes, which makes it difficult to improve the resistance only based on selection of few QTLs or marker-assisted selection (Omondi et al., 2023). However, the observed wide range of heritability estimates from low to high in multiple populations (**Table 1**) coupled with identification of consistent QTLs across populations suggests that NCLB resistance is predominantly controlled by additive effects. Several studies on genetic analyses of NCLB resistance reported the predominance of additive effects over non-additive effects (Vivek et al., 2010; Sibiya et al., 2013; Badu-Apraku et al., 2021). These results favor improving resistance through recurrent selection. Traditional recurrent selection is time-consuming and resource-intensive, and, on the other hand, genomic selection that captures all variations from small to large effects is well for such kind of traits to improve effectively. In tropical maize, genomic selection has been used for various economically important traits (Gowda et al., 2015, 2021; Ndlovu et al., 2022; Kimutai et al., 2023; Ndlovu et al., 2024b). Predictions within a population for NCLB resistance are moderate to high (0.42 to 0.55), which is comparable to earlier reported prediction accuracies by Omondi et al. (2023). Having an independent training population like historical data or related population data and being able to predict the breeding populations is more desirable for routine application of genomic selection in breeding. Here, we tried to predict individual DH populations by combining all DH populations as a training set. The prediction accuracies are moderate (r = 0.32 to 0.49) but comparable to the square root of the heritability estimates, which is equivalent to phenotypic selection efficiency (Lorenzana and Bernardo, 2009; Kibe et al., 2020a; Omondi et al., 2023). In the tropics, the opportunity to complete three cycles per year for genomic selection enables breeders to achieve high selection gain per year. Even if we are interested in predicting genotype performance in specific environments, it is important to include additional information from other environments, which help to improve the prediction accuracy. The marker by environment effect did not show much improvement in genome prediction compared to average prediction correlations and their standard deviations obtained in both CV1 and CV2 scenario (**Table 5**). This kind of model performs better in conditions in which the genotype-by-environment effect is large and marker effects are different in different environments. Overall, our results suggest that it is useful to have a common training population to predict NCLB resistance in multiple linked but distinct maize populations. This also helps the breeder to improve NCLB resistance and use their resources optimally in developing multiple stress-tolerant lines and hybrids.

## Conclusion

To dissect the genetic basis of NCLB resistance, we used linkage mapping, JLAM, GWAS, and genomic selection on the IMAS panel and biparental-based DH and F_3_ populations evaluated in multiple locations in Kenya. Linkage mapping in six biparental populations identified several minor- and major-effect QTLs with few overlapping across populations. JLAM identified 37 QTLs associated with NCLB resistance and many of them are co-located within the QTL detected in individual populations. Using 337,110 high-quality SNPs, GWAS identified 15 marker–trait associations. The putative candidate genes identified in the study are directly or indirectly involved in plant defense responses. However, their proposed functions require further validation to confirm the involvement of these genes in NCLB resistance. Several genomic regions were identified, which were found to be overlapping across different mapping approaches and with earlier studies. These genomic regions can serve as a potential target to improve CLB resistance. Genomic selection is a powerful methodology in plant breeding; however, its implementation is challenging because predictive model behavior depends on the specific conditions in which genome selection is used. Our results demonstrated that phenotypic selection to improve NCLB resistance under high disease pressure can be successfully supported by incorporating genomic selection in the ongoing breeding programs. Furthermore, significant variations in NCLB resistance observed across several populations imply that combining various sources of resistant alleles can be instrumental in increasing the levels of NCLB resistance in tropical maize.

## Data Availability

The datasets presented in this study can be found in online repositories. The names of the repository/repositories and accession number(s) can be found in the article/**Supplementary Material**. The markers used in this study were also used in our earlier study Kibe et al., 2020a; data used in this study can be found at https://data.cimmyt.org/dataset.xhtml?persistentId=hdl%3A11529%2F10548467.

## Supplementary material

Supplementary Table 1 *, ** significant at *P*< 0.05 and 0.01, respectively; LSD, least significant difference at 5%, CV, coefficient of variation; KKM, Kakamega; KIT, Kitale.

Supplementary Table 2 Genotypic correlations among locations for NCLB resistance in IMAS AM panel and six biparental populations. *, ** significant at *P*< 0.05 and 0.01, respectively; KKM, Kakamega; KIT, Kitale.
